# Depressive Symptoms, Emergency Care, and School Climate: An Ecological Analysis of Linked Administrative and Survey Data in New York City

**DOI:** 10.1111/josh.70014

**Published:** 2025-05-01

**Authors:** Sophia D. Arabadjis, Kira L. Argenio, Sophia E. Day, Kevin Konty, Stuart H. Sweeney

**Affiliations:** ^1^ Department of Geography University of California Santa Barbara Santa Barbara California USA; ^2^ Institute for Implementation Science and Population Health, City University of New York New York New York USA; ^3^ New York City Department of Health and Mental Hygiene Office of School Health New York New York USA; ^4^ Department of Epidemiology and Biostatistics City University of New York New York New York USA

**Keywords:** adolescent mental health, depression, education, emergency care, school health, YRBS

## Abstract

**Background:**

As rates of depression and anxiety continue to rise, prevention, and treatment of poor mental health in adolescents is a major challenge for population health. Within the US context, a growing body of literature is examining the relationship between school climate and student mental health.

**Methods:**

We extend the notion of school climate and mesosystem effects by creating an emergency department visit intensity, a novel indirect summary measure related to both the prevalence of depressive symptoms and district policy/resources. The intensity measure is linked to 3 years of the New York City Youth Risk Behavior Survey (2015, 2017, 2019), and we test the intensity measure in three models sequentially constructed from individual‐, school‐, and district‐level covariates across survey years with district‐level fixed effects.

**Results:**

We find strong evidence for a relationship between the prevalence of self‐reported depressive symptoms and individual and school climate indicators across all years, and document large differences in the prevalence of depressive symptoms and ED utilization by sex.

**Implications for School Health Policy, Practice, and Equity:**

Within the US context, adolescents spend much of their days in a school environment; our findings suggest that interventions could focus on female adolescents' experiences as well as the school (and district) to improve school climate and depressive symptoms indirectly.

## 1 Introduction

Studies conducted prior to the COVID‐19 pandemic revealed rising rates of depression and suicidal ideation in adolescents across the United States [1, 2, 3, 4, 5]. Estimates from the National Survey on Drug Use and Health suggest that the prevalence of a major depressive episode increased by 7.7% between 2009 and 2019 [1]. Evidence from the National Youth Risk Behavior Survey indicates that the prevalence of suicidal ideation has increased from 6.3% in 2009 to 18.8% in 2019 [3, 4]. Trends from the Monitoring the Future yearly surveys have found that depression trends while decreasing from 1991 to 2011, rebounded since and continued to increase through 2018 [5].

The increasing prevalence was not evenly distributed across subgroups; adolescent girls seem to be particularly affected and reported higher rates of depression, suicidal ideation, and suicide attempts across a range of surveys [1, 2, 5, 6, 7, 8, 9, 10]. At the same time, estimates of care provision for mental health over the same period remained steady at 19.7%, whereas the composition shifted to more visits by adolescent girls, more visits for suicidal ideation and depressive symptoms, and a decrease in care provided by school counselors nationally [10]. These trends paint a worrying picture for mental health among adolescents.

A growing body of research suggests that elements of the psychosocial environment of the school may be associated with rates of depression in the adolescent populations [11, 12, 13, 14, 15, 16]. These elements may include measures of bullying/victimization, perceptions of belonging, perceptions of school safety, having a trusted adult at school, and school disciplinary structures among others [11, 14, 15, 17, 18]. Together, these elements are often enveloped into measures of “school climate,” or “the quality and character of school life” [19]. As per the 2007 National School Climate Council, “school climate is based on patterns of students', parents' and school personnel's experience of school life; it also reflects norms, goals, values, interpersonal relationships, teaching and learning practices, and organizational structures” [11, 17, 19].

Although more work needs to be done to understand exact mechanisms linking school climate and mental health [13], researchers commonly draw on Bronfenbrenner's [20] (bio‐)ecological model of adolescent development. This conceptual model nests individuals within microsystems, mesosystems, and exosystems. The microsystem encompasses the direct face‐to‐face interactions of the adolescent, for example, an adolescent and their peers or guardians. The mesosystem is a level higher and links different actors in the microsystem, for example, interactions between guardians or peers. The outermost ring is the exosystem, the structures and institutions that guide/influence the mesosystems and microsystems directly experienced by the adolescent.

In this paper, we extend the notion of school climate by creating an emergency department (ED) visit intensity measure, a novel mesosystem summary measure related to both the prevalence of depressive symptoms and school policy and resources. Constructed at the district‐level, this measure combines data from a statewide ED and hospitalization database and school‐level demographic data to capture the rate at which students enter a hospital ED with a mental health‐related visit. The district ED intensity measures the rate at which students enter the hospital system within school hours during the academic year. Our assumption is that a sizeable portion of these students are being sent directly from school. Variation in the intensity among districts could reflect differences in student compositions, differences in school capacity and/or resource allocation, as well as differences in district policy and climate. We assess the ED intensity statistically in a series of regression models for individual depression rates that also include individual and school climate covariates.

## 2 Methods

The New York City Department of Health and Mental Hygiene (NYC DOHMH) Institutional Review Board determined this study to be public health surveillance and therefore “deemed to not be research” (45 CFR 46.102.l.2) and exempt from the requirement for obtaining written informed consent.

### 2.1 Data Sources

#### 2.1.1 Statewide ED Visits and Hospitalizations

The Statewide Planning and Research Cooperative System (SPARCS) was designed as an all‐payer collection of inpatient discharges for reimbursement, research, and planning purposes in New York State [21]. It has expanded to collect patient‐level characteristics, diagnoses, treatments, services, and charges for inpatient and outpatient services across the state. The SPARCS data for this study is limited to children and adolescents visiting the ED with a diagnosis code that qualifies under the Child and Adolescent Mental Health Disorders Classification System (CAMHD‐CS). There are 2390 unique codes from two different classification systems (ICD‐9, ICD‐10). The NYC DOHMH maintains a matched dataset that links SPARCS ED visits to records in the New York City Department of Education (NYC DOE) public schools' student enrollment data. From this matched dataset, we have limited covariates including the date‐of‐birth, the associated CAMHD‐CS ICD code, the visit date and the visit hour, student sex (M/F), the school identifier, and the student unique ID. More information on the matching process and covariates is available from the Office of School Health, Department of Health and Mental Hygiene.

**FIGURE 1:**
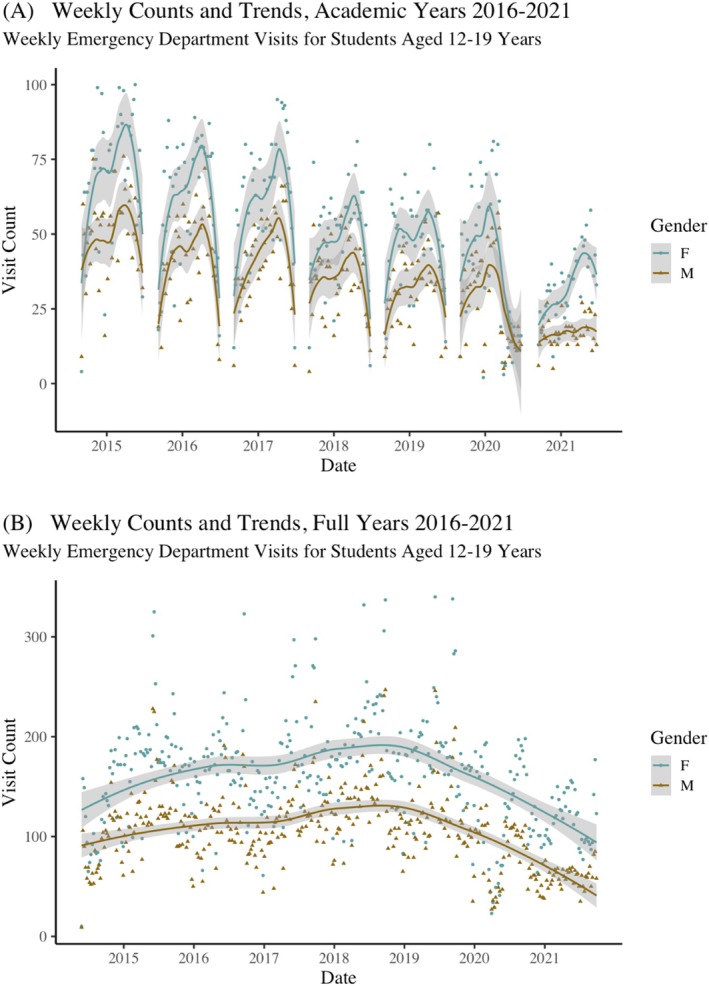
Weekly counts and trends: Figure 1A displays the weekly mental‐health related visits for students aged 12‐19 years that occured within school hours (7am to 4pm) excluding holidays (and summer vactions) and weekends. Figure 1B displays the weekly mental health related visits for students aged 12‐19 years over the entire period (no time, holiday or day‐of‐week restrictions.) Visit counts for males and females are starkly different; female students appear much more frequently in the ED for mental health‐related diagnoses than male students. Even restricted to the academic year and visits during school hours (A), the difference endures.

From the raw data we calculate age‐at‐visit (in years) and district (embedded in the school identifier). We subset the data to students aged 12–19 at visit, which corresponds to the Youth Risk Behavior Survey (YRBS) age sample and further limit the data to visits that occurred during the academic school year anytime from 7 a.m. to 4 p.m., excluding holidays and weekends. The start of the academic year generally occurs in September and runs through June; we use exact start and stop dates for each year. Trends in weekly visit counts and sex differences for the 5‐year period from 2014 to 2021 are shown in Figure 1 for the academic year (A), and the same period without the time restrictions on the visits (B). There are a total of 101,983 unique visits during this period for students aged 12–19, and just under a quarter (*n* = 24,251) occurred within school hours. We use only the academic year SPARCS data (as displayed in Figure 1A) in the subsequent analyses.

Note that the raw data used to produce this publication was purchased from or provided by the New York State Department of Health (NYSDOH). However, the calculations, metrics, conclusions derived, and views expressed herein are those of the author(s) and do not reflect the conclusions or views of NYSDOH. NYSDOH, its employees, officers, and agents make no representation, warranty, or guarantee as to the accuracy, completeness, currency, or suitability of the information provided here.

#### 2.1.2 School‐Level Demographic Data

New York City Open Data hosts a large collection of freely available data [22], such as school‐level demographic characteristics including grade enrollment counts and number/percent female enrollment, which is maintained by the NYC Department of Education [23]. Annual data for 2013–2021 were combined and summarized to produce yearly district‐level characteristics including total enrollment and enrollment by sex (counts and percents). Operational definitions of these covariates are described in the Supporting Information.

From 2014 to 2021, there were on average 325,037 students enrolled each year in Grades 9–12 across the 33 New York City public school districts. District enrollment ranges from around 1,353 to 36,707 students over the period. The three largest districts contributed approximately 10%, 5%, and 5% of students annually. Districts 75 and 79 were excluded from subsequent analyses because they are not part of the YRBS sampling frame. Though district 84 is comprised of charter schools across NYC, it is included in the YRBS sampling frame, so it was included for analysis.

#### 2.1.3 Youth Risk Behavior Survey

The purpose of the Center for Disease Control's YRBS is to measure priority health‐risk behaviors that contribute to the leading causes of morbidity, mortality, and social problems among youth. It is specifically designed to monitor six types of priority health‐risk behaviors, including (1) behaviors that contribute to unintentional injuries and violence, (2) sexual behaviors that contribute to HIV infection, other STDs, and unintended pregnancy, (3) tobacco use, (4) alcohol and other drug use, (5) unhealthy dietary behaviors, and (6) physical inactivity. There are two separate sampling strategies for the YRBS: a 3‐stage strategy that yields nationally representative estimates of high school aged students' behaviors as well as a 2‐stage cluster sampling strategy (schools, classrooms) that supports inference at submetropolitan scales [24]. Conducted jointly by the NYC Department of Health and Mental Hygiene (DOHMH) and the NYC Department of Education (DOE), we use the New York City large urban school district YRBS sample (NYC YRBS) from years 2015, 2017, and 2019 in this analysis, which is based on the 2‐stage sampling strategy.

The NYC DOHMH and NYC DOE are permitted to modify the standard questionnaire prior to data collection so long as two‐thirds of the questions remain unchanged and response categories are limited. As such, there are questions asked in some years that are not asked in other years. After data collection, a standard 3‐stage weighting procedure including nonresponse adjustment, selection probability, and the post‐stratification factor is performed to make the sample representative of New York City's public high school‐aged student population [25, 26]. Response rates must be above 60% for surveys to be weighted.

To measure depressive symptoms, the NYC YRBS has one survey item that is commonly used: “During the past 12 months, did you ever feel so sad or hopeless almost every day for two weeks or more in a row that you stopped doing some usual activities?” Responses are “yes,” “no” or “missing” [2, 9, 15]. This question is available every year (2015, 2017, 2019) and has a reasonable missingness rate each year (2.8%, 5.9%, and 3.3%, respectively).

Consistent with prior literature in the field [2, 9, 15], we use several other items related to individual characteristics to investigate population‐level differences in depressive symptoms. Because the questions asked vary by year, not all questions are included in every year of analysis. When available, we include an indicator of sex, coded “male,” “female,” and “NA” as “other,” an indicator of sexual orientation, gender identity, grade, age, home language, unstable household, unstable sleeping place, counseling, and suicidal ideation. Two additional questions asked about food security in the 2019 survey were included in the analysis for that year.

To measure school‐climate perceptions, we use truancy related to safety concerns at school, threats of violence or actual injury received at school, carrying a weapon at school (2015, 2017 only), bullying on school property, electronic bullying, school cohesion (2019 only), and trusted adult at school (2019 only), as consistent with prior work [4, 7, 8, 11]. Tables S2 and S3 summarize the questions, response categories, and availability year‐to‐year.

### 2.2 Statistical Analysis

We hypothesize that district‐level differences in student ED use for mental health‐related visits could be related to school climate and individual likelihood of reporting depressive symptoms in several ways. Additionally, we hypothesize that both individual characteristics and school climate perceptions will be strongly related to the likelihood of reporting depressive symptoms. Hence, our statistical analysis is 2‐fold. First, we describe the construction of the district ED visit intensity measure as a novel indicator of school climate that meaningfully summarizes the dynamic (longitudinal) visit data into a single annual value. Second, we link the ED Visit intensity to the NYC YRBS at the district‐level and describe our modeling approach to understand the relationship between individual characteristics, school climate, and the likelihood of reporting depressive symptoms.

To construct the ED visit intensity, we combine the weekly counts of ED visits by district with the district demographic data. Conceptually, we use a rate where the numerator is the count of ED visits per week and the denominator is the enrollment per district. Although we explored using a direct estimate, we decided that the smoother modeled rate better reflected the underlying variation among districts; the modeled rate was more conservative than the direct estimate.

We used an overdispersed Poisson regression to recover the rate parameter, λ.

$$y_{i}∼\text{Poisson}\left(\mu_{i},\lambda_{i},\omega \right)$$

where $y_{i}$ is the count of visits per week, $\mu_{i}$ is the district‐year‐sex–specific enrollment count ($\log\left(\mu_{i}\right)$ is the offset), ω is the dispersion parameter estimated from the data, and $\lambda_{i}$ is estimated by an over‐parameterized model that captures all combinations of district, sex, and year with a nonparametric smoothing spline for the *i*‐th week of the year.

$$\ln\left(\lambda_{i}\right)=\beta_{1}\mathrm{se}x_{i}+\beta_{2}\mathrm{yea}r_{i}+\beta_{3}\text{distric}t_{i}+s\left(x_{i}\right)$$

The model fits sex, year, and district counts for the *i*‐th week count, and $\hat{\beta }$ are summed and exponentiated to get the unique sex–year–district intensity. A natural smoothing spline with three knots is used to capture the shape of the trend data and help with the visible temporal autocorrelation in Figure 1. To fit with the YRBS data, we also create an “other” category for sex that is a mean of the sex‐specific intensities for each district and year.

To test the effects of individual characteristics, school climate, and ED visit intensity, we link the intensities to the NYC YRBS survey at the district‐level and use a logistic regression specification where the outcome of interest is the probability of reporting depressive symptoms. With the exception of the ED visit intensity, all covariates included in the logistic regressions are items from the NYC YRBS survey. For each year of the NYC YRBS survey, we fit four stepwise logistic regression models. The first model is unadjusted and includes only the district ED intensity and district fixed effects on the right‐hand side. The second model includes only the individual characteristics as covariates. The third model expands the second model to include covariates that reflect perceptions of school climate. The final model builds on the third model and adds the district ED visit intensity.

The choice of covariates included in the final models was guided by the bivariate associations available in Figures S4–S6, consistency across years, multicollinearity concerns, and the missingness rate for different items. Of particular note, the indicator for unstable housing has a large non‐response or missing component (32% in 2015, 30% in 2017, and 29% in 2019 for the unweighted responses). However, because the NYC YRBS is notably missing measures of personal and familial resources or capital, we decided to keep this measure. Especially for survey years 2015 and 2017, this question is one of the few indicators associated with this domain. Additionally, models specified without the unstable home variable retain similar estimates and directions for other covariates. Fixed effects for district were also included in every model.

To compare model fits and covariate contributions, we used an analysis of variance with a nested Wald test as recommended for complex survey designs and all models were fit using the survey.glm suite for complex survey designs in R version 3.4.2 [25, 27, 28]. We set α=0.05 as our significance cutoff. Data and code versioning were managed with GitHub and templates for reproducible research [29].

Finally, we assessed how much the NYC DOHMH survey weights were affecting depression estimates using the variance inflation factor (VIF) [25]. The general guidelines are that the VIF should be under 4 [25, 30]; the calculated VIF for each survey year in this study met that criterion.

## 3 Results

**FIGURE 2:**
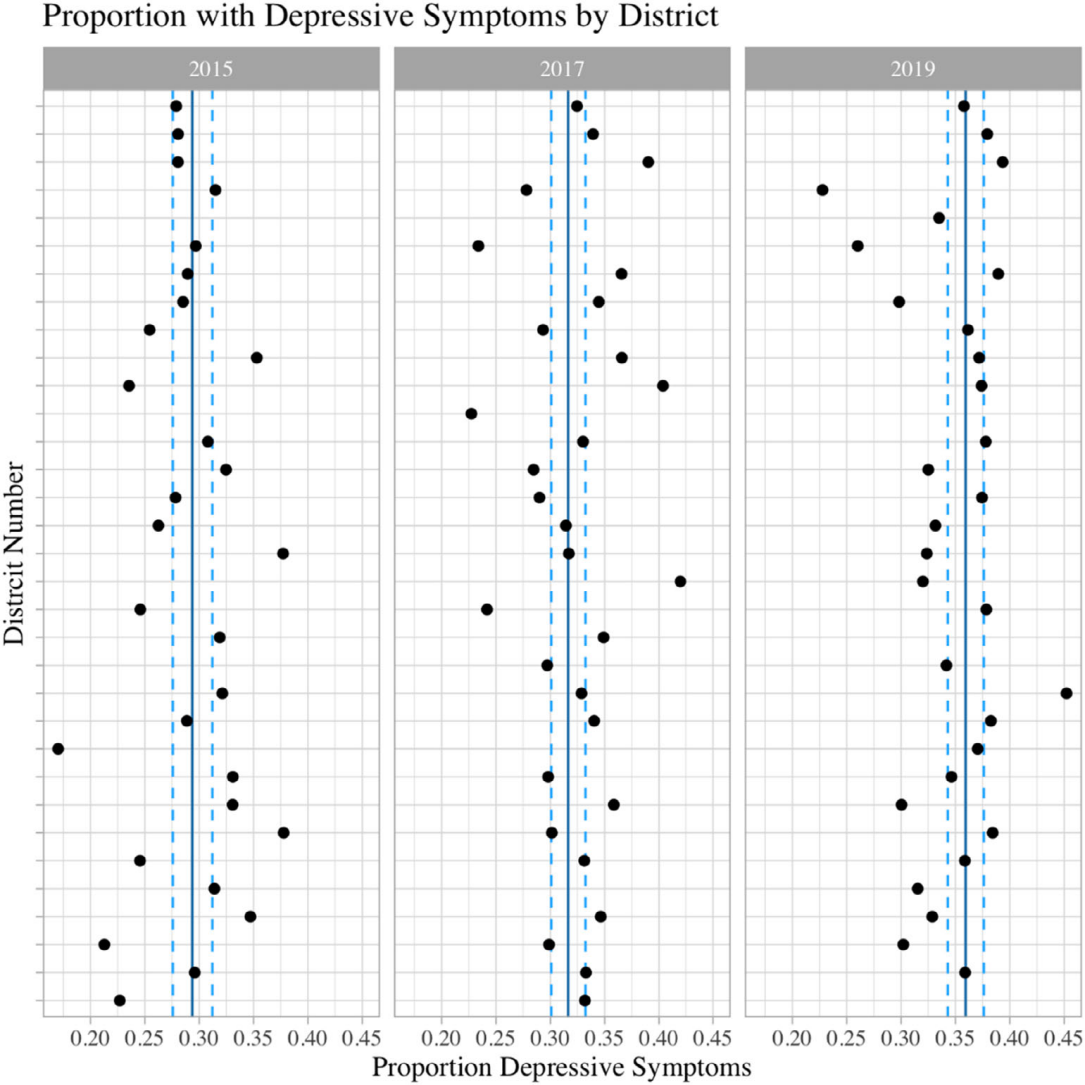
Proportion with depressive symptoms by district: The weighted proportion of students reporting depressive symptoms are displayed by district. The overall weighted proportion is displayed as a dark blue line, with associated confidence intervals in light blue. Confidence intervals of the population weighted district estimates are not calculable because of the weighting scheme (sparse strata). The survey weights are provided by NYC DOHMH.

Table 1 shows selected sample characteristics (weighted and unweighted) for the 2015, 2017, and 2019 surveys. Across all years, the proportion of students reporting depressive symptoms is above 25%. In 2015, 29.4% (95% CI [27.6, 31.0]) were estimated to have experienced depressive symptoms; in 2017, 31.6% [30.1, 33.0] and in 2019, 35.9% [34.3, 38.0] (displayed graphically in Figure 2). As shown in Figure 2, there was variation in the proportion of students reporting depressive symptoms across districts. Although the proportion of adolescents reporting depressive symptoms rose in each survey year (Figure 2), the overall ED visit counts during school hours fell (Figure 1A).

**TABLE 1:** Summary statistics: For each year of the YRBS sample, selected summary statistics are presented here in weighted and unweighted percentages (the exception being age, which is the sample mean).

Variable	2015		2017		2019	
	Unweighted %	Weighted %	Unweighted %	Weighted %	Unweighted %	Weighted %
Mean age (years)	15.62	15.45	15.5	15.45	15.54	15.44
Depression	29.0	29.4	30.2	31.6	34.7	35.9
Grade						
Grade 9	23.7	29.9	25.8	28.4	25.0	28.5
Grade 10	24.7	26.6	28.3	26.7	23.5	26.1
Grade 11	23.2	21.8	21.4	22.8	25.4	23.0
Grade 12	27.0	21.4	23.0	21.5	24.3	22.0
Ungraded/other	0.5	0.4	0.4	0.5	0.5	0.5
Sexual Orientation						
Bisexual	7.5	7.9	7.8	7.4	10.1	9.8
Gay or lesbian	2.3	2.0	3.0	3.0	3.0	3.3
Heterosexual	83.2	85.1	72	74.7	77.9	80.6
Not sure	5.0	5.0	13.9	14.9	6.1	6.4
Race						
Asian	11.1	16.8	11.9	16.5	13.3	13.4
Black	23.0	29.9	21.9	28.1	23.4	27.7
Hispanic	41.7	37.8	43.5	38.9	43.1	38.4
Other	6.2	2.1	5.5	2.3	4.9	5.9
White	12.5	13.4	12.0	14.2	10.5	14.5
Sex						
Female	50.6	48.8	50.9	48.2	51.0	48.6
Male	48.6	50.5	48	50.5	47.4	49.9
Other	0.8	0.7	1.1	1.2	1.6	1.6
Suicidal ideation	13.5	13.7	15.3	16.2	14.9	15.6
Transgender identity						
I do not understand the question	2.5	2.8	—	—	—	—
I don't know	1.1	1.2	—	—	—	—
Not transgender	91.4	93.3	90.6	98.4	51.9	76.0
Transgender	2.9	2.8	1.5	1.6	16.0	24.0
Unstable home	5.9	7.8	6.6	9.2	8.0	12.1
Borough						
Bronx	25.2	22.7	25.7	23.1	28.8	22.0
Brooklyn	28.3	31.2	29.6	30.4	24.6	29.5
Manhattan	10.3	11.1	11.6	9.3	11.5	10.7
Queens	21.3	28.1	19.1	30.0	20.2	30.3
Staten Island	14.9	6.8	12.1	7.3	14.8	7.4

*Note:* The percentages are similar between sample statistics and the weighted statistics for all the selected indicators, except for suicidal ideation (particularly in 2019) and New York City Borough. District sampling statistics are not shown for confidentiality reasons. Importantly for subsequent analyses, percentage reporting depressive symptoms is relatively even in the raw and weighted sample and increasing in prevalence across years. A full summary table with weighted and unweighted proportions and counts for each variable is presented in Table S4. Bivariate associations are also displayed visually in Figures S4–S6.

The unadjusted models show a significant relationship between district ED intensity, district fixed effects, and the likelihood of reporting depressive symptoms for 2015, 2017, and 2019. All associations were significant, though the magnitudes varied. Consider two districts; one has a district‐ED intensity of around one student per 10,000, whereas the other has a district‐ED intensity at the mean (around two students per 10,000). If a given student were to switch from the low‐ED intensity district to the high‐ED intensity district, our model suggests a significant increase in their probability of reporting depressive symptoms. In 2015, this increase would be 4.5%; in 2017, 6.6%; and in 2019, 13.3%.

**FIGURE 3:**
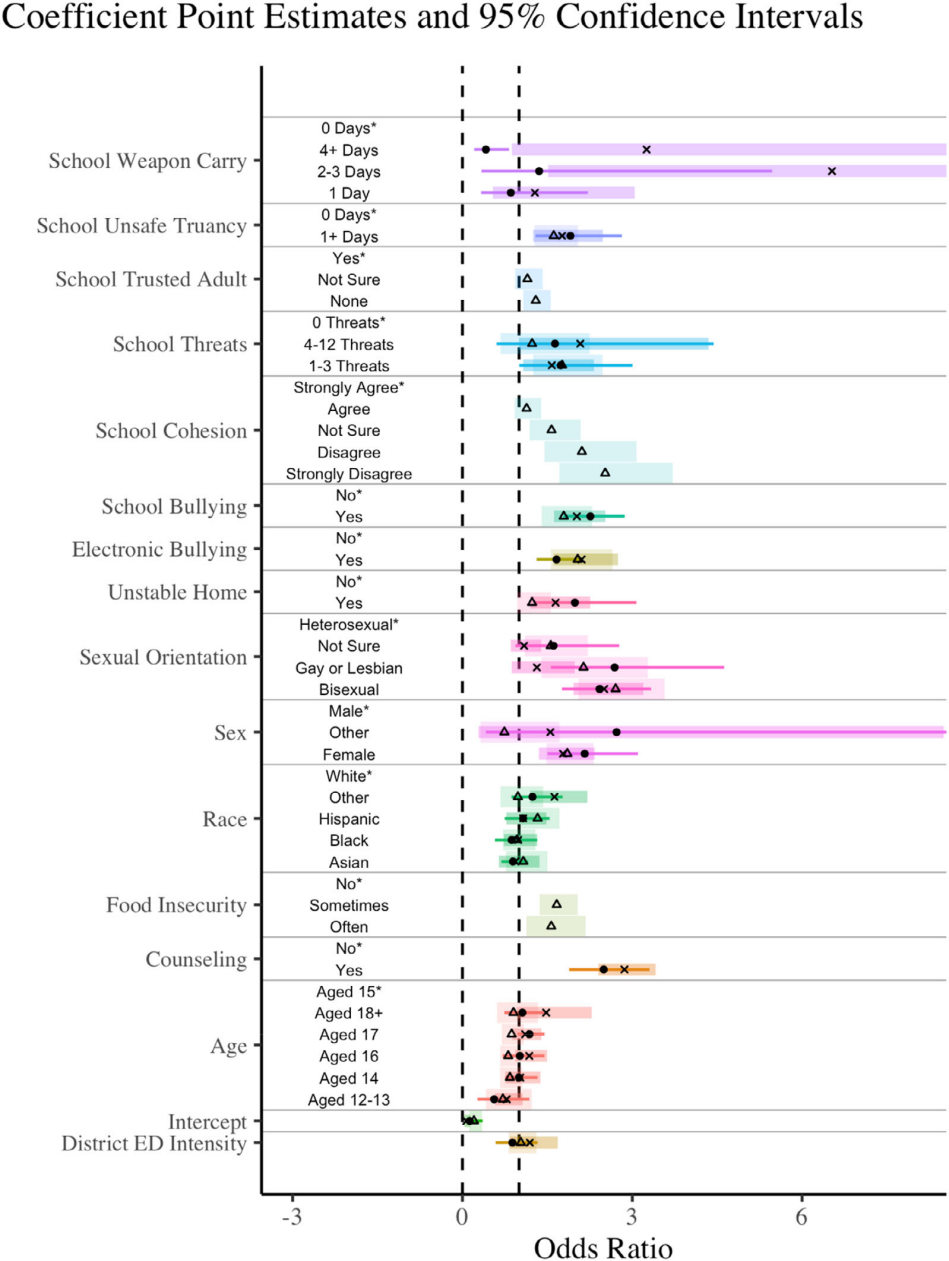
Coefficient plot: The model estimates for the full models across all years are displayed visually with their respective 95% confidence intervals. Points, *x*'s, and triangles represent point estimates from the 2015, 2017, and 2019 data years, respectively. The 95% confidence intervals are shown for each point estimate and increase in width to correspond with each associated year. The 95% confidence intervals that extend past 7 are truncated for visualization. Point estimates with confidence intervals that fall between 0 and 1 (dotted lines) are protective against reporting depressive symptoms; points and intervals that fall above 1 increase the risk of reporting depressive symptoms. Starred labels are reference categories.

Figure 3 displays the odds ratios (exponentiated log‐odds) and 95% confidence intervals for the final model specifications, including the individual and school climate perceptions and ED visit intensity covariates. Values greater than 1 indicate an increase in the associated odds; values between 0 and 1 indicate a decreased likelihood of reporting depressive symptoms. The final model specifications and log‐odds estimates are given in Tables S5–S7.

In all models, across all years, female sex is strongly associated with an increase in the probability of reporting depressive symptoms, echoing the raw ED visit trends of Figure 1. In 2015, the odds of having depressive symptoms are 1.9–2.4 times higher for females than for male students, depending on the model specification and holding all other indicators in their reference category. Although we tried multiple specifications interacting sex with sexual orientation, age, and race, none improved model fit or residual diagnostics.

The models with only individual characteristics revealed somewhat similar patterns across years; sex, sexual orientation, counseling in the past 12 months, and unstable housing are consistently associated with an increasing probability of depressive symptoms. Further analyses of variance for each year suggests that age and race are never a significant covariate at α=0.05 (not shown).

In the models with both individual characteristics and perceptions of school climate covariates, the significant individual covariates tend to maintain the direction of their associations but change slightly in magnitude. An exception to this is the sexual orientation covariate. In 2015 and 2019, all categories relative to heterosexual were significantly associated with an increase in the likelihood of depressive symptoms. In 2017, students identifying as gay or lesbian, or bisexual were more likely to report depressive symptoms per the model estimates, but not when perceptions of school climate were included (only bisexual retained its significant association). In the 2019 models, food insecurity was significantly associated with an increase in the odds of reporting depressive symptoms.

Poor perceptions of school climate, including safety‐based truancy, receiving threats of violence or injury on school grounds, carrying a weapon to school, and all forms of bullying, are associated with a significant increase in the odds of reporting depressive symptoms. Similarly, in 2019, not having a trusted adult at school and poor perceptions of school cohesion were associated with a significant increase in the likelihood of reporting depressive symptoms. Results of nested Wald tests suggest that inclusion of the perceptions of school climate significantly improved overall model fit.

In the unadjusted models for each year, the ED intensity was significantly associated with the likelihood of reporting depressive symptoms, but this was likely due to the large differential between intensities for males versus females in any given district (additional model specifications confirmed this finding). In the full models, the district ED intensity measure was not significantly associated with the likelihood of reporting depressive symptoms. However, the district fixed effects were consistently associated with an increase in the probability. The relationship between the district fixed effects and the ED intensity is conceptually challenging. Although both measures are indicative of mesosystem pathways, further investigation suggests they may be reflecting different processes. The fixed effects are only moderately correlated with the ED intensities each year (0.16 in 2015, 0.11 in 2017, and − 0.34 in 2019). Similarly, in a regression framework only 3%–12% of the observed variation in the ED intensities can be attributed to the fixed effect coefficients ($R^{2}=0.03,\;0.01,\;\text{and}\;0.12$ in 2015, 2017, and 2019, respectively).

## 4 Discussion

Across the literature, individual indicators like sex, sexual orientation, and substance use [1, 2, 5, 9, 15, 31], microsystem relationships with peers and adults [15], and mesosystem perceptions of school‐climate [11, 12, 13] have been consistently associated with adolescent depression. This growing body of evidence suggests that individual, microsystem, and mesosystem effects exist in the socio‐spatial context of adolescent depression and mental health. In this study, we developed a novel candidate measure designed to reflect mesosystem differences in school climate based on ED visit patterns across school districts. We hypothesized that this measure of ED intensity could reflect differences in district student composition, differences in district resources for mental health, and differences in school climate (e.g., district disciplinary policy), though the measure itself would not distinguish between these pathways.

Our results suggest that sex is a particularly important factor in adolescent mental health—both in the likelihood of reporting depressive symptoms as well as in emergency healthcare utilization. In particular, female sex elevated the risk of reporting depressive symptoms substantially in all of our models, which accords well with prepandemic trend analyses [1, 2, 5]. Additional individual characteristics including sexual orientation, counseling, and unstable home were consistently and significantly associated with increased risk, whereas age and race/ethnicity were not in any of our models. Perceptions of school climate including experience of bullying, threats at school, and truancy related to personal safety were all consistently associated with an increase in the risk of reporting depressive symptoms across all models and years. Of particular interest, Seil and coauthors published results using the 2009 NYC YRBS to examine the co‐occurrence of mental health, school trusted adult, substance use, and differences in sexual orientation. Our results extend these findings to newer data and are weighted to be representative of the NYC high school‐aged population.

Our novel measure of ED intensity does not seem to capture anything new at the mesosystem scale. This could be for a variety of reasons, both conceptual and methodological. The ED intensity is potentially better positioned at the school level as opposed to the district level to capture our hypothesized pathways. It could also be that these hypothesized pathways need to be better delineated with additional controls, such as the presence of a school counselor or local Neighborhood Health Action Center. Finally, expanding from the ecological model to socio‐spatial determinants of health, there are many other spatially varying covariates that may be associated with both school climate and individual propensity for reporting depressive symptoms, such as an adolescent's perception of their school's built environment [15, 32, 33]. Although these differences may be reflected in the district fixed effects, they are perhaps too distal to be reflected in the ED visit intensity. Still, the consistent and significant association between the district fixed effects and the symptoms of adolescent depression suggests unexplained variation at the mesosystem of the ecological model. Though mildly correlated, our measure of ED intensity does not seem to explain much of that remaining variation.

There are several limitations to this study. From a context standpoint, mental health care was rapidly changing for this 5‐year period. In 2015, Mayor De Blasio launched ThriveNYC (now the Mayor's Office of Community Mental Health), which was intended to overhaul New York City's mental health system. A continuing goal for the program is to reduce reliance on ED utilization and make care available earlier and in the community, particularly for youth populations [34, 35]. Coupled with the onset of the COVID‐19 pandemic, we could not disentangle changing healthcare access from ThriveNYC or pandemic restrictions during this period. From a statistical standpoint, ideally we would have been able to implement a hierarchical logistic regression model that accounted for the complex survey design. In that setting, including a group‐level covariate (such as an ED intensity) and a fixed effect (district) could reduce the unexplained between‐group variation and provide more insight. Constrained to nested models, the relationship between ED intensity and depressive symptom reporting given school and individual characteristics remains unclear.

Additionally, our descriptive results suggest opposing trends: Although the proportion of students reporting depressive symptoms rose with every subsequent survey year, the count of mental health‐related ED visits during school hours fell from 2014 to 2021. This discrepancy has several possible explanations, including changes in policy during the period (ThriveNYC), changes in district‐level resources or trends (nursing, counseling, policing in schools, Neighborhood Health Action Centers), and changing correlation between depressive symptoms and ED visits. Our study research is limited in its ability to explore or test such pathways, but coupled with our findings of significant district fixed effects, additional research in these areas with additional administrative data may be fruitful.

This study also has several strengths. We use 3 years of YRBS survey data that has been weighted to be representative of the NYC public high school‐aged adolescent population, which allows us to infer the population prevalence of depressive symptoms over time. Additionally, we show one approach to link dynamic hospitalization and ED visit data to surveys to provide additional insights. Finally, this study extends the ecological model to show clear ecological effects, which, with future work, could suggest that interventions on school climate may be beneficial in lowering the prevalence of depressive symptoms. In particular, the unexplained variation at the district level warrants additional research and, as we have shown in this paper, novel indicators constructed from administrative data could help researchers disentangle mesosystem effects for place‐based interventions. These efforts should continue, and future work could extend this approach to examine school‐level ED intensities, age‐specific ED intensities, or ED intensities of residential neighborhoods (to identify high ED utilizing communities). Future modeling work could also encompass more explicit measures of district or school resources and student body composition to control for those hypothesized pathways.

## 5 Conclusion

Our results have implications for researchers, public health practitioners, and school staff and administrators. For researchers, we describe an extension of the ecological model that relates micro‐ and mesosystem factors to adolescent mental health. For public health practitioners, our results demonstrate the research potential of linked administrative and survey data. If a survey can be re‐identified (such as at the district level), then additional data sources can be used to bolster analyses—especially in areas where survey data may not be sufficient. For school staff and administrators in New York City, whereas ED visits for mental health fell between 2015 and 2019, depressive symptoms continued to climb, especially for adolescent women. Our results suggest school climate is consistently and significantly associated with the reporting of depressive symptoms; interventions to improve school climate may help overall adolescent mental health.

## Conflicts of Interest

The authors declare no conflicts of interest.

## Supporting information


**Data S1.** josh70014‐sup‐0001‐Supinfo.

## Data Availability

The data that support the findings of this study are available from the New York State Department of Health. Restrictions apply to the availability of these data, which were used with IRB approval for this study. Data are available with the permission of the New York State Department of Health for approved studies.
