# Supplementary material for: Depressive Symptoms, Emergency Care, and School Climate: An Ecological Analysis of Linked Administrative and Survey Data in New York City
Source: J Sch Health. 2025 May 1;95(6):441–50. doi: 10.1111/josh.70014 (PMC12088930; doi:10.1111/josh.70014)
Supplement: Supplementary file 1 — Data S1. josh70014‐sup‐0001‐Supinfo. [file JOSH-95-441-s001.pdf]

Appendices

Label	Year	Local Alias	Definition	Domain	Missingness
Age	2015	NQ1	(12 = 1,13,...,18+ = 7)	Individual	46/8522 (0.5%))
Orientation	2015	NQ68	(Heterosexual (straight) = 1, Gay or lesbian, Bisexual, Not Sure = 4)	Individual	172/8522 (2.0%)
Transgender	2015	NQ93	(Not trans = 1, yes transman, yes transwoman, yes transother, I don't know, I don't know what this question is asking = 6)	Individual	175/8522 (2.1%)
Sex	2015	NQ2 (Q2)	(Female = 1, Male)	Individual	71/8522 (0.8%)
Grade	2015	NQ3 (Q3)	(9th = 1, 10th, 11th, 12th, Ungraded/Other = 5)	Individual	89/8522 (1.04%)
Race5	2015	...	(Asian, Black, Hispanic, White, Other)	Individual	472/8522 (5.5%)
Home Language	2015	NQ92	How often is a language other than English spoken at home; (Never,=1 Rarely, Sometimes, Most of the time, Always = 5)	Individual	132/8522 (1.5%)
Counseling	2015	NQ95	During the past 12 months, (Yes = 1, No = 2)	Individual	512/8522 (6.0%)
Unstable Sleep	2015	NQ121 (Q95)	During the past 30 days, (parents/guardian's home, friends family lost home, motel or hotel, shelter or emergency housing, car park campground or public place, move, somewhere else)	Individual	2517/8522 (29.5%)
Unstable Home	2015	NQ122 (Q96)	During the past 12 months, ever live away from parents/guardians (Yes, No)	Individual	2718/8522 (31.9%)
Depression	2015	NQ26	In past 12 months, depressive episode; (Yes = 1, No = 2)	Outcome	240/8522 (2.8%)
Suicide	2015	NQ27	In past 12 months, considered suicide attempt; (Yes = 1, No = 2)	Outcome	283/8522 (3.3%)
School Bullying	2015	NQ24	In past 12 months, bullied on school property; (Yes, No)	Perceptions	179/8522 (2.1%)
Electronic Bullying	2015	NQ25 (Q25)	In past 12 months, electronically bullied; (Yes, No)	Perceptions	210/8522 (2.5%)
School Unsafe Truancy	2015	NQ16 (Q19)	In past 30 days, days skipped school because felt unsafe; (0 days = 1, 1, 2-3, 4-5, 6+ = 5)	Perceptions	210/8522 (2.5%)
School Threat	2015	NQ17 (Q20)	In past 12 months, threatened/injured on school property with weapon; (0 times, 1, 2-3, 4-5, 6-7,8-9,10-11,12+ = 8)	Perceptions	69/8522 (0.8%)
School Weapon Carry	2015	NQ15 (Q19)	In past 30 days, times carried a weapon on school property; (0 days, 1, 2-3, 4-5, 6+ = 5)	Perceptions	210/8522 (2.5%)
Age	2017	NQ1 (Q1)	(12,13,...,18+)	Individual	53/10191 (0.5%)
Orientation	2017	NQ67 (Q15)	(Heterosexual (straight), Gay or lesbian, Bisexual, Not Sure)	Individual	331/10191 (3.2%)
Transgender	2017	NQ95 (Q13)	Are you transgender; (No, Yes, Not sure, I don't understand)	Individual	320/10191 (3.1%)
Sex	2017	NQ2 (Q2)	Sex; (Female, Male)	Individual	114/10191 (1.1 %)
Grade	2017	NQ3 (Q3)	(9th, 10th, 11th, 12th, Ungraded/Other)	Individual	114/10191 (1.1%)
Race5	2017		(White, Black, Hispanic, Asian, Other)	Individual	531/10191 (5.2%)
Home Language	2017	NQ93 (Q11)	How often is a language other than English spoken at home; (Never, Rarely, Sometimes, Most of the time, Always)	Individual	77/10191 (0.8%)
Counseling	2017	NQ98 (Q32)	During the past 12 months, (Yes, No, Not Sure)	Individual	574/10191 (5.6%)
Unstable Sleep	2017	NQ122 (Q93)	During the past 30 days, (parents/guardian's home, friends family lost home, motel or hotel, shelter or emergency housing, car park campground or public place, no usual place, somewhere else)	Individual	2839/10191 (27.9%)
Unstable Home	2017	NQ123 (Q94)	During the past 12 months, ever live away from parents/guardians (Yes, No)	Individual	3104/10191 (30.5%)
Depression	2017	NQ25 (Q28)	In past 12 months, depressive episode; (Yes, No)	Outcome	610/10191 (6.0%)
Suicide	2017	NQ26 (Q30)	In past 12 months, considered suicide attempt; (Yes, No)	Outcome	498/10191 (4.9%)
School Bullying	2017	NQ23 (Q25)	In past 12 months, bullied on school property; (Yes, No)	Perceptions	328/10191 (3.2%)
Electronic Bullying	2017	NQ24 (Q26)	In past 12 months, electronically bullied; (Yes, No)	Perceptions	241/10191 (2.3%)
School Unsafe Truancy	2017	NQ15 (Q19)	In past 30 days, days skipped school because felt unsafe; (0 days, 1, 2-3, 4-5, 6+)	Perceptions	291/10191 (2.9%)
School Threat	2017	NQ17 (Q21)	In past 12 months, threatened/injured on school property with weapon; (0 times, 1, 2-3, 4-5, 6-7,8-9,10-11,12+)	Perceptions	277/10191 (2.7%)
School Weapon Carry	2017	NQ13 (Q18)	In past 30 days, times carried a weapon on school property; (0 days, 1, 2-3, 4-5, 6+)	Perceptions	417/10191 (4.1%)

Table 2: 2015 and 2017 YRBS questions considered for bivariate analysis.

Label	Year	Local Alias	Definition	Domain	Missingness
Counseling	2019	...	...	...	Not Asked
Age	2019	NQ1 (Q1)	(12,13,...,18+)	Individual	49/9534 (0.5%)
Orientation	2019	NQ66 (Q66)	(Heterosexual (straight), Gay or lesbian, Bisexual, Not Sure)	Individual	274/9534 (2.9%)
Transgender	2019	NQ95 (Q13)	Are you transgender; (No, Yes, Not sure, I don't understand)	Individual	240/9534 (2.5%)
Sex	2019	NQ2 (Q2)	Sex; (Female, Male)	Individual	152/9534 (1.6%)
Grade	2019	NQ3 (Q3)	(9th, 10th, 11th, 12th, Ungraded/Other)	Individual	128/9534 (1.3%)
Race5	2019	...	(White, Black, Hispanic, Asian, Other AI/AN Hawaiian PI Multiple)	Individual	462/9534 (4.8%)
Home Language	2019	NQ91 (Q91)	How often is a language other than English spoken at home; (Never, Rarely, Sometimes, Most of the time, Always)	Individual	141/9534 (1.5%)
Food Insecurity A	2019	NQ121 (Q121)	In past 12 months, how often worried food would run out before money to buy more; (Often, Sometimes, Never)	individual	2524/9534 (26.5%)
Food Insecurity B	2019	NQ122 (Q122)	In past 12 months, how often did food run out before money to buy more; (Often, Sometimes, Never)	individual	2577/9534 (27.0%)
Unstable Sleep	2019	NQ119 (Q119)	During the past 30 days, (parents/guardian's home, friends family lost home, foster or group home, motel or hotel, shelter or emergency housing, car park campground or public place, no usual place, somewhere else)	Individual	2334/9534 (24.5%)
Unstable Home	2019	NQ120 (Q120)	During the past 12 months, ever live away from parents/guardians (Yes, No)	Individual	2758/9534 (29.0%)
Depression	2019	NQ25 (Q25)	In past 12 months, depressive episode; (Yes, No)	Outcome	315/9534 (3.3%)
Suicide	2019	NQ26 (Q26)	In past 12 months, considered suicide attempt; (Yes, No)	Outcome	340/9534 (3.6%)
School Cohesion	2019	NQ125 (Q125)	Agree/Disagree close with people at school; (Strongly Agree, Agree, not sure, disagree, strong disagree)	Perceptions	2526/9534 (26.5%)
School Trusted Adult	2019	NQ126 (Q126)	Have a teacher/adult at school trust; (Yes, No, Not Sure)	Perceptions	2692/9534 (28.2%)
School Bullying	2019	NQ23 (Q23)	In past 12 months, bullied on school property; (Yes, No)	Perceptions	260/9534 (2.7%)
Electronic Bullying	2019	NQ24 (Q24)	In past 12 months, electronically bullied; (Yes, No)	Perceptions	279/9534 (2.9%)
School Unsafe Truancy	2019	NQ15 (Q15)	In past 30 days, days skipped school because felt unsafe; (0 days, 1, 2-3, 4-5, 6+)	Perceptions	104/9534 (1.1%)
School Threat	2019	NQ16 (Q16)	In past 12 months, threatened/injured on school property with weapon; (0 times, 1, 2-3, 4-5, 6-7,8-9,10-11,12+)	Perceptions	57/9534 (0.6%)
School Weapon Carry	2019	...	In past 30 days, times carried a weapon on school property; (0 days, 1, 2-3, 4-5, 6+)	Perceptions	Not Asked

Table 3: 2019 YRBS questions considered for bivariate analysis.

Variable	2015 Unweighted	2015 Weighted	2017 Unweighted	2017 Weighted	2019 Unweighted	2019 Weighted
Mean Age	15.619	15.45	15.504	15.448	15.541	15.438
Age: Missing	36	-	53	-	49	-
	(0.004)	-	(0.005)	-	(0.005)	-
Counseling	1450	47391	1798	47144	-	-
	(0.17)	(0.183)	(0.176)	(0.191)	-	-
Counseling: Missing	512	-	1135	-	-	-
	(0.06)	-	(0.111)	-	-	-
Depression	2469	78785	3078	82774	3311	94627
	(0.29)	(0.294)	(0.302)	(0.316)	(0.347)	(0.359)
Depression: Missing	240	-	610	-	315	-
	(0.028)	-	(0.06)	-	(0.033)	-
Electronic Bullying	1022	32475	1319	35835	1323	37843
	(0.12)	(0.121)	(0.129)	(0.133)	(0.139)	(0.143)
Electronic Bullying: Missing	210	-	241	-	279	-
	(0.025)	-	(0.024)	-	(0.029)	-
Food Insecure: Never	-	-	-	-	5469	148325
	-	-	-	-	(0.574)	(0.777)
Food Insecure: Often	-	-	-	-	558	16194
	-	-	-	-	(0.059)	(0.085)
Food Insecure: Sometimes	-	-	-	-	930	26496
	-	-	-	-	(0.098)	(0.139)
Food Insecure: Blank	-	-	-	-	2577	-
	-	-	-	-	(0.27)	-
Food Insecure Worry: Never	-	-	-	-	5078	138889
	-	-	-	-	(0.533)	(0.721)
Food Insecure Worry: Often	-	-	-	-	728	21303
	-	-	-	-	(0.076)	(0.111)
Food Insecure Worry: Sometimes	-	-	-	-	1204	32373
	-	-	-	-	(0.126)	(0.168)
Food Insecure Worry: Missing	-	-	-	-	2524	-
	-	-	-	-	(0.265)	-
Grade 9	2016	81695	2625	77979	2383	76604
	(0.237)	(0.299)	(0.258)	(0.284)	(0.25)	(0.285)
Grade 10	2101	72599	2883	73184	2244	70037
	(0.247)	(0.266)	(0.283)	(0.267)	(0.235)	(0.261)
Grade 11	1976	59500	2181	62621	2418	61641
	(0.232)	(0.218)	(0.214)	(0.228)	(0.254)	(0.23)
Grade 12	2301	58380	2349	59010	2318	58949
	(0.27)	(0.214)	(0.23)	(0.215)	(0.243)	(0.22)
Ungraded/Other	39	992	39	1312	43	1209
	(0.005)	(0.004)	(0.004)	(0.005)	(0.005)	(0.005)
Grade Missing	89	-	114	-	128	-
	(0.01)	-	(0.011)	-	(0.013)	-
HL: Always English	1955	62286	2245	62806	1978	60606
	(0.229)	(0.23)	(0.22)	(0.228)	(0.207)	(0.226)
HL: Always not English	2024	65899	2614	70475	2575	71985
	(0.238)	(0.243)	(0.257)	(0.256)	(0.27)	(0.268)
HL: Mostly not English	2010	66493	2401	65016	2262	62218
	(0.236)	(0.245)	(0.236)	(0.236)	(0.237)	(0.232)
HL: Rarely not English	745	24288	934	25877	858	24686
	(0.087)	(0.089)	(0.092)	(0.094)	(0.09)	(0.092)
HL: Sometimes not English	1656	52413	1920	50867	1720	48673
	(0.194)	(0.193)	(0.188)	(0.185)	(0.18)	(0.182)
HL: Missing	132	-	77	-	141	-
	(0.015)	-	(0.008)	-	(0.015)	-

Table 4: This table summarizes the weighted and unweighted descriptive statistics for each YRBS sample year. Proportions are presented in parentheses, and look relatively similar before and after weighting.

	2015		2017		2019	
Variable	Unweighted	Weighted	Unweighted	Weighted	Unweighted	Weighted
Orientation: Bisexual	637	21150	792	19870	966	25696
	(0.075)	(0.079)	(0.078)	(0.074)	(0.101)	(0.098)
Orientation: Gay or Lesbian	195	5419	309	8127	290	8568
	(0.023)	(0.02)	(0.03)	(0.03)	(0.03)	(0.033)
Orientation: Heterosexual	7089	227875	7340	200945	7425	212165
	(0.832)	(0.851)	(0.72)	(0.747)	(0.779)	(0.806)
Orientation: Not Sure	429	13259	1419	40068	579	16747
	(0.05)	(0.05)	(0.139)	(0.149)	(0.061)	(0.064)
Orientation: Missing	172	-	331	-	274	-
	(0.02)	-	(0.032)	-	(0.029)	-
Race: Asian	947	43538	1212	43265	1264	34712
	(0.111)	(0.168)	(0.119)	(0.165)	(0.133)	(0.134)
Race: Black	1957	77641	2227	73621	2233	71632
	(0.23)	(0.299)	(0.219)	(0.281)	(0.234)	(0.277)
Race: Hispanic	3552	97986	4438	101842	4109	99157
	(0.417)	(0.378)	(0.435)	(0.389)	(0.431)	(0.384)
Race: Other	532	5388	556	5901	466	15350
	(0.062)	(0.021)	(0.055)	(0.023)	(0.049)	(0.059)
Race: White	1062	34867	1227	37220	1000	37584
	(0.125)	(0.134)	(0.12)	(0.142)	(0.105)	(0.145)
Race: Missing	472	-	531	-	462	-
	(0.055)	-	(0.052)	-	(0.048)	-
School Bullying	1210	39979	1506	41287	1534	45218
	(0.142)	(0.148)	(0.148)	(0.155)	(0.161)	(0.171)
School Bullying: Missing	179	-	328	-	260	-
	(0.021)	-	(0.032)	-	(0.027)	-
School Cohesion: Agree	-	-	-	-	3054	82627
	-	-	-	-	(0.32)	(0.429)
School Cohesion: Disagree	-	-	-	-	610	17063
	-	-	-	-	(0.064)	(0.089)
School Cohesion: Not Sure	-	-	-	-	1416	39841
	-	-	-	-	(0.149)	(0.207)
School Cohesion: Strongly Agree	-	-	-	-	1555	43458
	-	-	-	-	(0.163)	(0.226)
School Cohesion: Strongly Disagree	-	-	-	-	373	9607
	-	-	-	-	(0.039)	(0.05)
School Cohesion: Missing	-	-	-	-	2526	-
	-	-	-	-	(0.265)	-
School Trusted Adult: No	-	-	-	-	1527	41698
	-	-	-	-	(0.16)	(0.222)
School Trusted Adult: Not sure	-	-	-	-	1065	28526
	-	-	-	-	(0.112)	(0.152)
School Trusted Adult:Yes	-	-	-	-	4246	117497
	-	-	-	-	(0.445)	(0.626)
School Trusted Adult: Missing	-	-	-	-	2696	-
	-	-	-	-	(0.283)	-
School Threats: 1-3	362	12677	495	14120	529	15831
	(0.042)	(0.046)	(0.049)	(0.052)	(0.055)	(0.058)
School Threats: 4-12	160	4635	222	6648	244	6717
	(0.019)	(0.017)	(0.022)	(0.025)	(0.026)	(0.025)
School Threats: 0	7931	255360	9197	250374	8704	248143
	(0.931)	(0.937)	(0.902)	(0.923)	(0.913)	(0.917)
School Threats: Missing	69	-	277	-	57	-
	(0.008)	-	(0.027)	-	(0.006)	-

Table 4 continued.

Variable	2015 Unweighted	2015 Weighted	2017 Unweighted	2017 Weighted	2019 Unweighted	2019 Weighted
School Unsafe Truancy: At least 1 day	535	15941	849	23258	951	27719
	(0.063)	(0.06)	(0.083)	(0.086)	(0.1)	(0.104)
School Unsafe Truancy: Missing	214	-	291	-	170	-
	(0.025)	-	(0.029)	-	(0.018)	-
School Weapon Carry: 0 days	8025	260275	9473	258369	-	-
	(0.942)	(0.969)	(0.93)	(0.967)	-	-
School Weapon Carry: 1 day	83	2504	84	2807	-	-
	(0.01)	(0.009)	(0.008)	(0.011)	-	-
School Weapon Carry: 2-3 days	65	1647	49	1293	-	-
	(0.008)	(0.006)	(0.005)	(0.005)	-	-
School Weapon Carry: 4+ days	139	4285	168	4589	-	-
	(0.016)	(0.016)	(0.016)	(0.017)	-	-
School Weapon Carry: Missing	210	-	417	-	-	-
	(0.025)	-	(0.041)	-	-	-
Sex: Female	4312	134368	5184	133732	4866	132177
	(0.506)	(0.488)	(0.509)	(0.482)	(0.51)	(0.486)
Sex: Male	4139	139111	4893	140174	4516	135674
	(0.486)	(0.505)	(0.48)	(0.505)	(0.474)	(0.499)
Sex: Other	71	2059	114	3423	152	4247
	(0.008)	(0.007)	(0.011)	(0.012)	(0.016)	(0.016)
Suicidal Ideation	1147	36548	1557	42902	3311	40982
	(0.135)	(0.137)	(0.153)	(0.162)	(0.347)	(0.156)
Suicidal Ideation: Missing	283	-	498	-	315	-
	(0.033)	-	(0.049)	-	(0.033)	-
Transgender Identity: I do not understand	212	7417	-	-	-	-
	(0.025)	(0.028)	-	-	-	-
Transgender Identity: I don't know	98	3141	-	-	-	-
	(0.011)	(0.012)	-	-	-	-
Transgender Idenity: Not transgender	7786	250136	9231	251903	4950	141025
	(0.914)	(0.933)	(0.906)	(0.984)	(0.519)	(0.76)
Transgender Identity: Yes	251	7536	150	4082	1530	44643
	(0.029)	(0.028)	(0.015)	(0.016)	(0.16)	(0.24)
Transgender Identity: Missing	175	-	810	-	3054	-
	(0.021)	-	(0.079)	-	(0.32)	-
Unstable Home: Yes	507	15330	671	17303	759	22565
	(0.059)	(0.078)	(0.066)	(0.092)	(0.08)	(0.121)
Unstable Home: Missing	2718	-	3104	-	2758	-
	(0.319)	-	(0.305)	-	(0.289)	-
Sleep Location: car, park, campground	26	711	25	672	23	490
	(0.003)	(0.004)	(0.002)	(0.003)	(0.002)	(0.002)
Sleep Location: Did not have usual place	-	-	-	-	25	645
	-	-	-	-	(0.003)	(0.003)
Sleep Location: Foster or Group Home	-	-	120	3507	178	5063
	-	-	(0.012)	(0.018)	(0.019)	(0.026)
Sleep Location: Hidden Homelessness	251	7390	349	9457	407	11698
	(0.029)	(0.037)	(0.034)	(0.048)	(0.043)	(0.059)
Sleep Location: Home	5532	187283	6697	177038	6400	175006
	(0.649)	(0.93)	(0.657)	(0.906)	(0.671)	(0.885)
Sleep Location: Motel or Hotel	80	1987	24	883	49	1604
	(0.009)	(0.01)	(0.002)	(0.005)	(0.005)	(0.008)
Sleep Location: Move from place to place	17	468	26	786	-	-
	(0.002)	(0.002)	(0.003)	(0.004)	-	-
Sleep Location: Shelter or Emergency housing	60	2224	72	1848	92	2531
	(0.007)	(0.011)	(0.007)	(0.009)	(0.01)	(0.013)
Sleep Location: Somewhere Else	39	1286	39	1249	26	795
	(0.005)	(0.006)	(0.004)	(0.006)	(0.003)	(0.004)
Sleep Location: Missing	2517	-	2839	-	2334	-
	(0.295)	-	(0.279)	-	(0.245)	-

Table 4 continued.

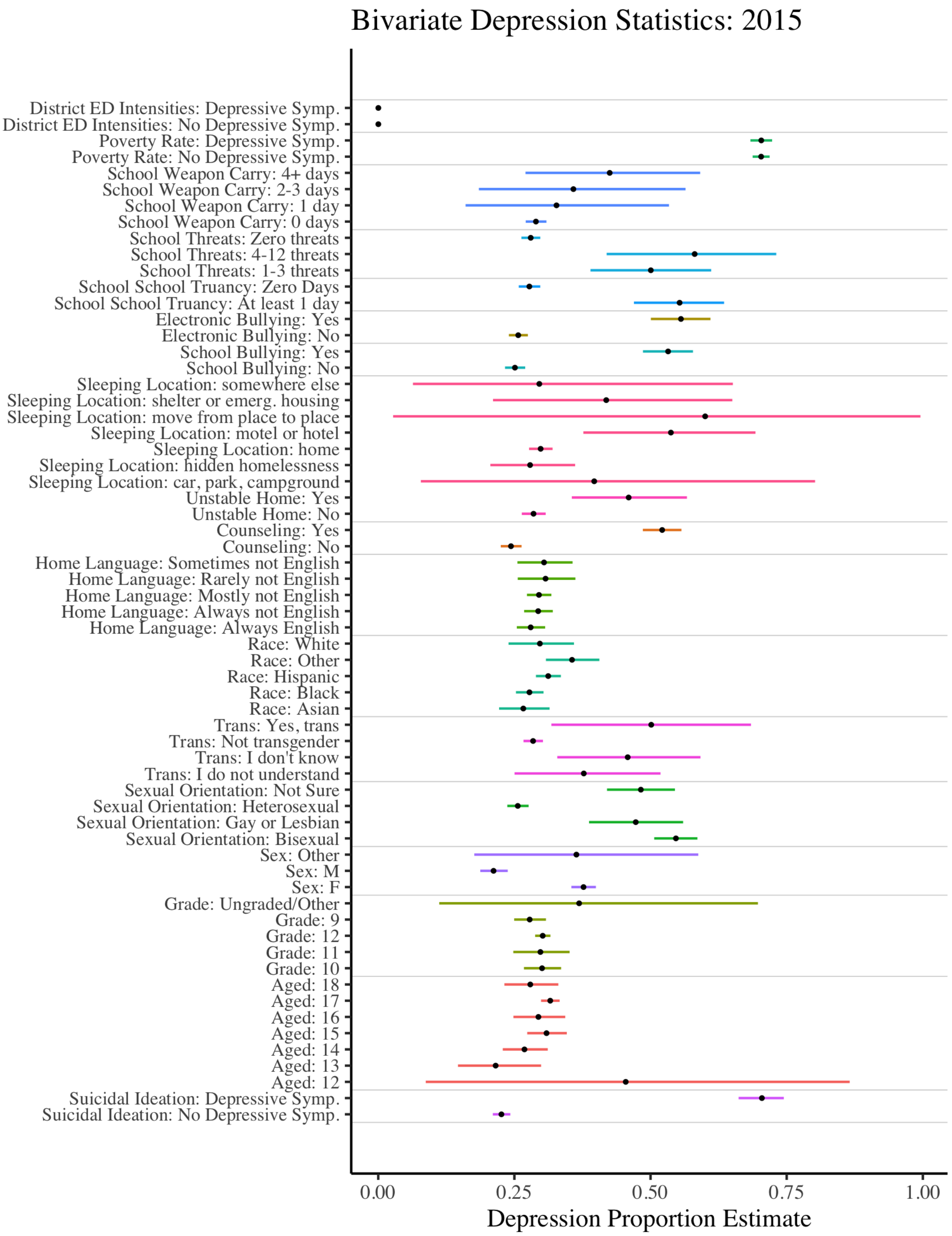

Figure 4: Point estimates and 95% CI for the bivariate associations between depression and various covariates for YRBS 2015.

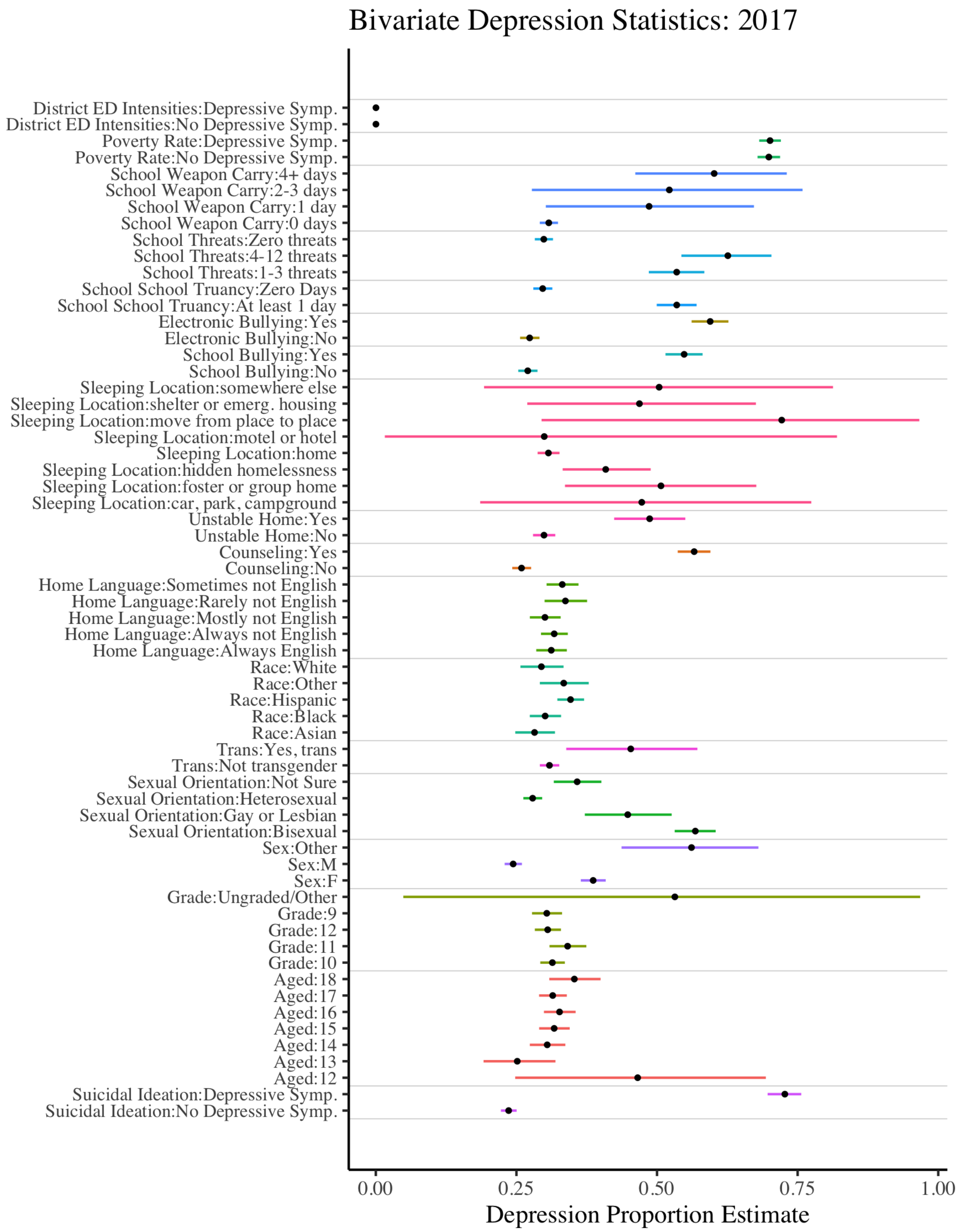

Figure 5: Point estimates and 95% CI for the bivariate associations between depression and various covariates for YRBS 2017.

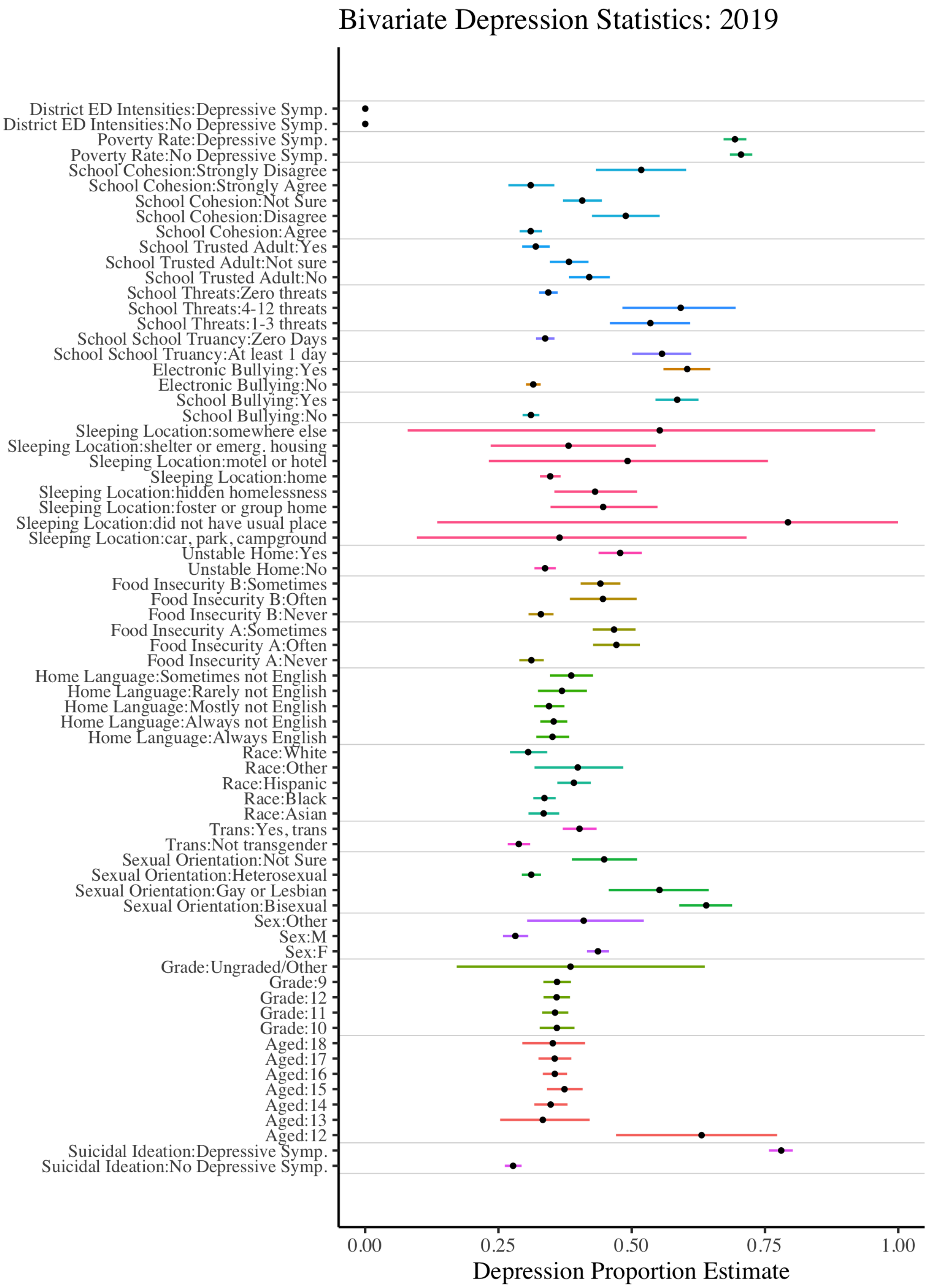

Figure 6: Point estimates and 95% CI for the bivariate associations between depression and various covariates for YRBS 2019.

	Dependent variable:		
	Depression Symptoms		
	(Ind)	(Ind + School)	(Ind + School + District)
Constant	-1.951^{***} (0.216)	-2.321^{***} (0.181)	-2.055^{***} (0.523)
Male			
Female	0.669^{***} (0.105)	0.687^{***} (0.099)	0.770^{***} (0.184)
Other (sex)	1.094 (1.029)	0.959 (0.919)	1.002 (0.960)
Age 12-13	-0.583^{*} (0.349)	-0.596 (0.387)	-0.581 (0.381)
Age 14	-0.036 (0.153)	-0.0001 (0.145)	-0.001 (0.145)
Age 15			
Age 16	-0.059 (0.169)	0.012 (0.182)	0.014 (0.182)
Age 17	0.075 (0.100)	0.167 (0.105)	0.170 (0.102)
Age 18+	-0.059 (0.154)	0.055 (0.182)	0.057 (0.183)
Heterosexual			
Bisexual	0.948^{***} (0.154)	0.884^{***} (0.163)	0.884^{***} (0.163)
Gay or Lesbian	0.868^{***} (0.216)	0.993^{***} (0.280)	0.989^{***} (0.277)
Not Sure	0.738^{***} (0.204)	0.480^{*} (0.277)	0.476^{*} (0.277)
No Counseling			
Counseling	1.015^{***} (0.138)	0.914^{***} (0.144)	0.915^{***} (0.143)
Stable Home			
Unstable Home	0.776^{***} (0.235)	0.687^{***} (0.222)	0.686^{***} (0.222)
White			
Asian	-0.140 (0.150)	-0.112 (0.131)	-0.114 (0.133)
Black	-0.296 (0.227)	-0.138 (0.210)	-0.140 (0.213)
Hispanic	-0.057 (0.194)	0.070 (0.184)	0.069 (0.185)
Other	0.164 (0.188)	0.218 (0.180)	0.213 (0.183)
No School Unsafe Truancy			
School Unsafe Truancy		0.643^{***} (0.198)	0.645^{***} (0.199)
School Threats: 0			
School Threats: 1-3		0.552^{*} (0.279)	0.552^{*} (0.280)
School Threats: 4-12		0.511 (0.509)	0.491 (0.510)
School Weapon Carry: 0 days			
School Weapon Carry: 1 day		-0.160 (0.489)	-0.157 (0.486)
School Weapon Carry: 2-3 days		0.295 (0.707)	0.303 (0.712)
School Weapon Carry: 4+ days		-0.888^{**} (0.345)	-0.882^{**} (0.350)
No School Bullying			
School Bullying		0.815^{***} (0.121)	0.815^{***} (0.121)
No Electronic Bullying			
Electronic Bullying		0.511^{***} (0.121)	0.508^{***} (0.121)
District ED Intensity			-0.125 (0.208)
District	*	*	*
Observations	5,209	5,048	5,048
Note:			*p<0.1; **p<0.05; ***p<0.01

Table 5: YRBS 2015 Depression Models

	Dependent variable:		
	Depression Symptoms		
	(Ind)	(Ind + School)	(Ind + School + District)
Constant	-1.857^{***} (0.228)	-2.295^{***} (0.152)	-2.680^{***} (0.374)
Male			
Female	0.611^{***} (0.055)	0.693^{***} (0.062)	0.574^{***} (0.140)
Other (sex)	0.562 (0.602)	0.488 (0.857)	0.440 (0.867)
Age 12-13	−0.143 (0.202)	−0.243 (0.159)	−0.246 (0.157)
Age 14	0.047 (0.172)	0.019 (0.153)	0.021 (0.152)
Age 16	0.106 (0.122)	0.164 (0.121)	0.165 (0.120)
Age 17	0.027 (0.116)	0.102 (0.117)	0.102 (0.117)
Age 18+	0.322 (0.201)	0.391^{*} (0.222)	0.391^{*} (0.222)
Heterosexual			
Bisexual	0.973^{***} (0.116)	0.921^{***} (0.125)	0.918^{***} (0.124)
Gay or Lesbian	0.454^{**} (0.202)	0.283 (0.208)	0.274 (0.210)
Not Sure	0.166 (0.127)	0.088 (0.124)	0.085 (0.124)
No Counseling			
Counseling	1.161^{***} (0.087)	1.054^{***} (0.089)	1.051^{***} (0.090)
Stable Home			
Unstable Home	0.641^{***} (0.161)	0.494^{***} (0.160)	0.497^{***} (0.161)
White			
Asian	−0.090 (0.178)	−0.064 (0.192)	−0.067 (0.191)
Black	−0.046 (0.148)	−0.016 (0.150)	−0.016 (0.150)
Hispanic	0.067 (0.157)	0.070 (0.164)	0.070 (0.165)
Other	0.437^{***} (0.139)	0.485^{***} (0.157)	0.486^{***} (0.156)
No School Unsafe Truancy			
School Unsafe Truancy		0.566^{***} (0.175)	0.565^{***} (0.175)
No School Threats			
School Threats: 1-3		0.452^{**} (0.193)	0.457^{**} (0.197)
School Threats: 4-12		0.740^{*} (0.379)	0.733^{*} (0.376)
School Weapon Carry: 0 days			
School Weapon Carry: 1 day		0.231 (0.441)	0.247 (0.442)
School Weapon Carry: 2-3 days		1.862^{**} (0.742)	1.876^{**} (0.746)
School Weapon Carry: 4+ days		1.170^{*} (0.670)	1.180^{*} (0.670)
No School Bullying			
School Bullying		0.702^{***} (0.114)	0.704^{***} (0.113)
No Electronic Bullying			
Electronic Bullying		0.743^{***} (0.135)	0.744^{***} (0.136)
District ED Intensity			0.174 (0.177)
District Fixed Effect	*	*	*
Observations	5,998	5,874	5,874
Note:			*p<0.1; **p<0.05; ***p<0.01

Table 6: YRBS 2017 Depression Models

	Dependent variable:		
	Depression Symptoms		
	(Ind)	(Ind + School)	(Ind + School + District)
Constant	−1.008*** (0.201)	−1.564*** (0.244)	−1.597*** (0.273)
Male			
Female	0.606*** (0.065)	0.640*** (0.064)	0.617*** (0.113)
Other (sex)	−0.315 (0.312)	−0.289 (0.426)	−0.300 (0.429)
Age 12-13	−0.206 (0.237)	−0.338 (0.275)	−0.337 (0.275)
Age 14	−0.102 (0.125)	−0.176 (0.118)	−0.176 (0.118)
Age 15			
Age 16	−0.209** (0.093)	−0.213** (0.096)	−0.213** (0.096)
Age 17	−0.157 (0.109)	−0.140 (0.112)	−0.140 (0.112)
Age 18+	−0.024 (0.163)	−0.103 (0.199)	−0.104 (0.199)
Heterosexual			
Bisexual	1.063*** (0.136)	0.996*** (0.142)	0.995*** (0.142)
Gay or Lesbian	0.962*** (0.217)	0.759*** (0.217)	0.760*** (0.217)
Not Sure	0.590*** (0.188)	0.444** (0.179)	0.445** (0.179)
Stable Home			
Unstable Home	0.309** (0.118)	0.207* (0.122)	0.207* (0.121)
White			
Asian	0.151 (0.168)	0.072 (0.170)	0.072 (0.170)
Black	−0.004 (0.143)	−0.039 (0.149)	−0.039 (0.149)
Hispanic	0.286** (0.123)	0.283** (0.130)	0.283** (0.130)
Other	0.063 (0.187)	−0.021 (0.191)	−0.021 (0.192)
Food Insecurity: Never			
Food Insecurity: Sometimes	0.636*** (0.093)	0.510*** (0.102)	0.510*** (0.102)
Food Insecurity: Often	0.507*** (0.157)	0.450*** (0.167)	0.450*** (0.166)
No School Unsafe Truancy			
School Unsafe Truancy		0.478*** (0.119)	0.478*** (0.120)
School Threats: 0			
School Threats: 1-3		0.566*** (0.174)	0.565*** (0.174)
School Threats: 4-12		0.207 (0.307)	0.208 (0.307)
No School Bullying			
School Bullying		0.582*** (0.126)	0.582*** (0.126)
No Electronic Bullying			
Electronic Bullying		0.710*** (0.134)	0.711*** (0.135)
School Trusted Adult: Yes			
School Trusted Adult: Not sure		0.138 (0.108)	0.137 (0.108)
School Trusted Adult: No		0.259*** (0.095)	0.259*** (0.095)
School Cohesion: Strongly Agree			
School Cohesion: Agree		0.125 (0.105)	0.125 (0.105)
School Cohesion: Not Sure		0.453*** (0.144)	0.453*** (0.144)
School Cohesion: Disagree		0.746*** (0.192)	0.747*** (0.192)
School Cohesion: Strongly Disagree		0.925*** (0.198)	0.925*** (0.197)
District ED Intensity			0.030 (0.120)
District Fixed Effect	*	*	*
Observations	6,200	5,883	5,883
Note:			*p<0.1; **p<0.05; ***p<0.01

Table 7: YRBS 2019 Depression Models

District-Level Variables: Operational Definitions & Notes

Of particular interest are the total enrollment, female enrollment, and number/percent impoverished. In general students with families that have qualified for free or reduced price meals or are eligible for Human Resource Administration benefits are considered impoverished. Further details of the poverty indicator are given by NYC Open Data (NYC DOE & NYC DOHMH, 2024b).While we investigated including number/percent impoverished in our model specifications; it never improved overall model fit or diagnostics.

- Total enrollment (sum of grade 9, 10, 11, and 12 students)
- Female enrollment (sum of grade 9, 10, 11, and 12 female students)
- Male enrollment (sum of grades 9, 10, 11 and 12 male students)
- Percent female enrollment (Female enrollment/total enrollment)
- Percent male enrollment (1 - percent female enrollment for rounding purposes)
- Poverty enrollment count (% poverty enrollment multiplied by total enrollment)[9]
- Percent poverty (poverty enrollment/total enrollment)

[9] The focus on this analysis is on high school-aged students, but some schools are K-12 institutions. These data do not contain poverty enrollment by grade, so I assume the overall percentage is equivalent for each grade in this preliminary analysis. While this may be a reasonable approximation, other indicators (such as the school's Economic Need Index) may be a good additional next step as this captures percentage of students facing economic hardship.
